# Helical reconstruction of amyloids in *cryoSPARC*

**DOI:** 10.1107/S2053230X26003675

**Published:** 2026-06-09

**Authors:** Jan-Hannes Schaefer, Robert T. O’Neill, Joseph P. Donnelly, Evan T. Powers, Jeffery W. Kelly, Gabriel C. Lander

**Affiliations:** a Department of Integrative Structural and Computational Biology Scripps Research Institute La Jolla California USA; b Department of Chemistry, Chi-Huey Wong Laboratories for Biomedical Research Scripps Research Institute La Jolla California USA; University of Leeds, United Kingdom

**Keywords:** amyloids, cryo-EM, helical reconstruction, *cryoSPARC*

## Abstract

Amyloid-mediated proteotoxicity underlies over 50 diseases. Cryo-EM establishes direct links between filament morphologies and pathology, although microbial functional amyloids illustrate that this fold can evolve to serve physiological roles, unlike their pathogenic counterparts. Despite the growing popularity of the processing software *cryoSPARC* for single-particle analyses, *RELION* remains the dominant software platform for performing helical reconstruction of amyloid structures, highlighting an area for further development. Here, we present comprehensive processing guidelines for helical reconstruction of helical amyloids using *cryoSPARC*. Through systematic reprocessing and validation of publicly deposited datasets, we demonstrate the current capabilities and identify the key limitations, emphasizing the need for amyloid-specific parameter optimization within *cryoSPARC* workflows. Our findings showcase a potential for developing unsupervised processing workflows to meet the demanding throughput requirements of time-resolved *in vitro* studies and large-scale compound-screening initiatives, thereby accelerating therapeutic drug development. Ultimately, our goal is to shift the focus of amyloid cryo-EM from computationally intensive processing challenges towards addressing fundamental biological questions that enhance our capacity for treatment discovery.

## Abstract 2

We present practical guidelines for helical reconstruction of amyloid filaments in *cryoSPARC*, benchmarked against publicly available datasets. Our analysis defines current capabilities and limitations, and outlines optimization strategies to enable high-throughput structural studies and therapeutic discovery.

## 1. Introduction

Many macromolecules assemble into filaments with helical symmetry to enable essential cellular functions, including motility, morphological organization and macromolecular trafficking (McManus *et al.*, 2016 ▸). While these universal assemblies typically increase protein stability and allow responsiveness to physiological changes through directed assembly and disassembly (Ghosal & Löwe, 2015 ▸), aberrant protein homeostasis can result in pathological fibril formation following irreversible conformational changes. The resulting amyloid assemblies are characterized by stable cross-β-strand arrangements and have a propensity to accumulate into deposits called plaques. Such plaques are implicated in over 50 human diseases, including Alzheimer’s disease (AD), Parkinson’s disease (PD) and transthyretin amyloidosis (ATTR) (Iadanza *et al.*, 2018 ▸; Knowles *et al.*, 2014 ▸).

While mostly disease-associated in humans, amyloids evolved as functional assemblies found in many microbial organisms. Following the secretion of amyloid precursor proteins, functional amyloids assemble into physical barriers against predators such as bacteriophages, provide structural support in biofilms or act as virulence factors (Akbey & Andreasen, 2022 ▸; Otzen & Riek, 2019 ▸).

Since amyloid fibrils are generally recalcitrant to crystallization and are too large for NMR analysis, cryo-electron microscopy (cryo-EM) has emerged as the primary method for resolving the molecular details of amyloidogenic disease pathologies. While today’s single-particle workflows can achieve true atomic resolution (Nakane *et al.*, 2020 ▸), helical reconstruction differs substantially from the traditional workflows used for single-particle analyses of macromolecules, requiring specialized processing procedures for accurate structure determination. Helical symmetry optimization tools were previously implemented in *RELION* to specifically target amyloids, which enabled the first reconstruction of patient-derived tau fibrils (Fitzpatrick *et al.*, 2017 ▸; He & Scheres, 2017 ▸). These initial studies led to a flurry of *ex vivo* amyloid structures, identifying a link between amyloid folds and underlying disease pathologies and accelerating our understanding of these devastating diseases (Otzen & Riek, 2019 ▸; Scheres *et al.*, 2023 ▸; Taylor & Staniforth, 2022 ▸).

**Figure 1:**
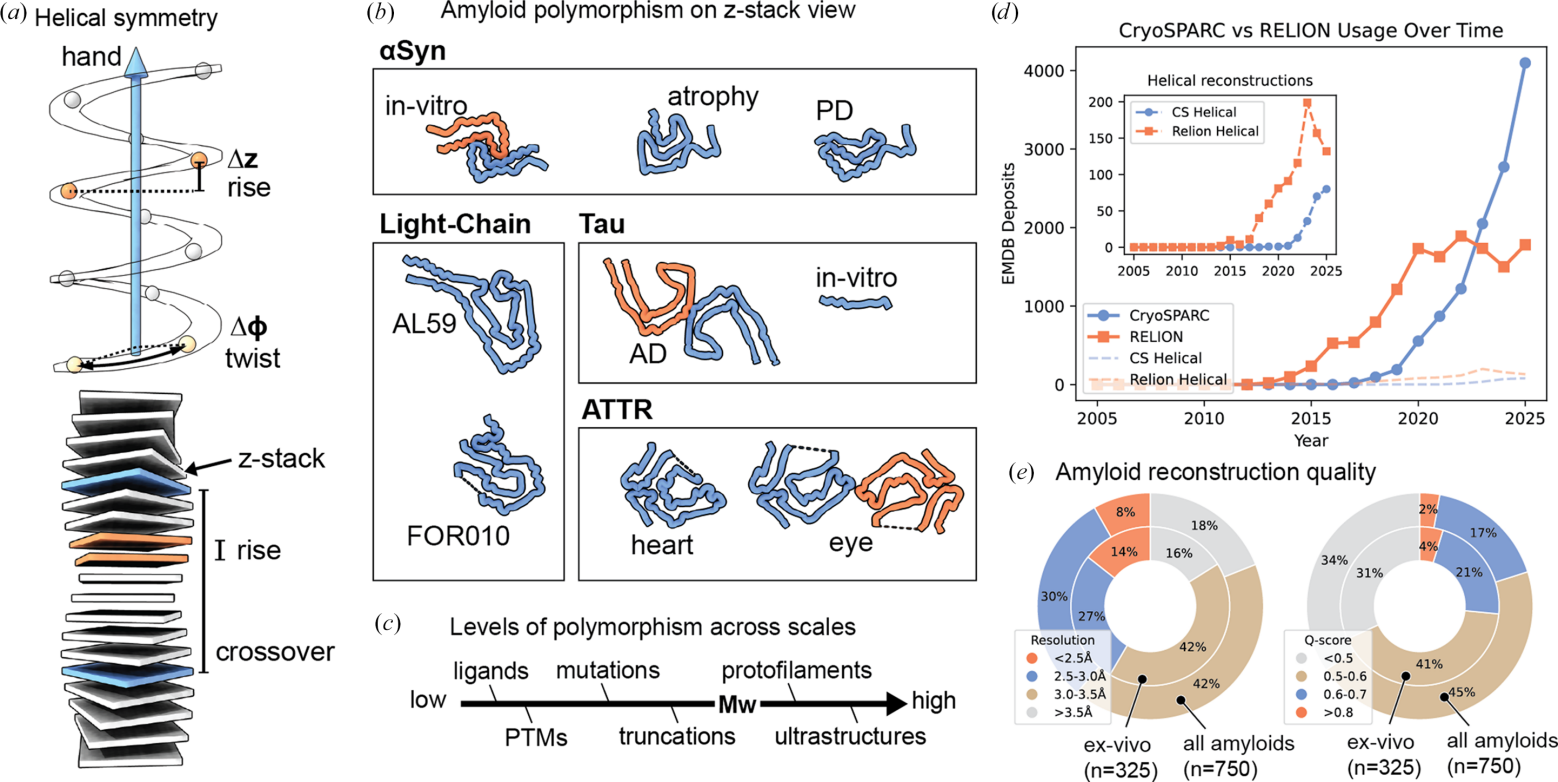
Architecture, diversity and reconstructions of amyloids. (*a*) Schematic representation of helical symmetry parameters: helical rise (Δ*z*, orange) and helical twist (Δφ, tan) with central helical *z* axis (blue). The arrow indicates helix handedness. Amyloid *z*-stack model showing rise (orange) and crossover distance (blue). (*b*) Cross-β-peptide arrangements of amyloid polymorphs grouped by amyloid protein. Single-chain polymorphs are shown in blue and double-chain polymorphs in orange/blue. AL59 and FOR010 represent patient identifiers from light-chain amyloidosis cases. (*c*) Contributors to amyloid polymorphism organized by molecular weight (Mw), including post-translational modifications (PTMs). (*d*) Cumulative EMDB depositions categorized by processing software: *RELION* (orange) and *cryoSPARC* (blue). Data for 2026 were omitted for annual comparison consistency. Helical reconstructions are highlighted in the inset. (*e*) Validation metrics for deposited amyloid reconstructions using Amyloid Atlas data. *Q*-scores and resolutions at FSC 0.143 for *ex vivo* and all annotated amyloid entries displayed as nested pie charts.

Amyloids present unique structural challenges, as they usually present as helical assemblies with constrained screw operators, requiring helical indexing over a shallow helical rise value range (Δ*z*) and narrow twist range (Δφ) (Fig. 1 ▸*a*). This distinctive symmetry, combined with the substantial structural polymorphism observed in *ex vivo* samples, makes automated filament picking and accurate initial model generation particularly challenging (Lövestam & Scheres, 2022 ▸). Additional complexity arises from substantial structural polymorphism (Fig. 1 ▸*b*). Differences in peptide packing result in polymorphs at the protofilament level, while ultrastructural differences arise from varying numbers of interacting protofilaments within the amyloid fibril. Compositional polymorphs result from ultrastructures of different amyloid proteins and non-amyloid components (Zielinski *et al.*, 2021 ▸). Post-translational modifications such as glycosylation, disulfide bridges in light-chain amyloids (Radamaker *et al.*, 2021 ▸) or α-synuclein phosphorylation in PD (Zhao *et al.*, 2020 ▸) add another level of heterogeneity (Fig. 1 ▸*c*). These structural complexities have necessitated amyloid-specific cryo-EM processing tools, which have been successfully developed and implemented within *RELION* (He & Scheres, 2017 ▸; Kimanius *et al.*, 2021 ▸; Lövestam *et al.*, 2024 ▸).

While the Electron Microscopy Data Bank (EMDB) currently reports 14 196 unique *RELION*-generated reconstructions, compared with approximately 13 421 unique reconstructions from *cryoSPARC* (Punjani *et al.*, 2017 ▸), most helical reconstructions, including nearly all amyloid structures, have been processed using *RELION* (https://www.ebi.ac.uk/emdb/emsearch/charts/; Fig. 1 ▸*d*). Of the 760 amyloid reconstructions documented in the Amyloid Atlas (Sawaya *et al.*, 2021 ▸), only bacterial PSMα1 polymorphs (EMD-43835; Hansen *et al.*, 2024 ▸), one *ex vivo* light-chain amyloid reconstruction (EMD-70557; Hicks *et al.*, 2026 ▸), catalytic amyloids from an anaerobic amoeboid organism (EMD-53305; Kunnath *et al.*, 2025 ▸), *Pseudomonas* FapC fibrils (EMD-49649; Hansen *et al.*, 2025 ▸) and a yet to be published amyloid of human RIPK1 (EMD-52356) have successfully been processed using *cryoSPARC*.

One likely reason for this discrepancy stems from a user’s greater ability to tightly control parameter optimization within *RELION*, whereas *cryoSPARC* prioritizes generalized processing guidelines.

Effective software development relies on community involvement and feedback. To expand *cryoSPARC* into a truly universal processing pipeline capable of handling both single-particle analysis and helical reconstruction, comprehensive guidelines for reconstructing amyloids need to be established and formalized.

This work aims to systematically evaluate the current capabilities of *cryoSPARC* (v.4.7) for resolving amyloid structures while identifying key limitations in existing processing workflows. We use five *RELION*-processed EMPIAR datasets as case studies and ground-truth references to cross-validate reconstructions generated within *cryo­SPARC*. Our comprehensive analysis guides users through the complete processing pipeline: from initial assessment of the collected micrographs and rational selection of helical symmetry parameters, through classification of heterogeneous samples and reconstruction of homogeneous polymorphs, to final validation using established community-driven metrics. By providing these systematic guidelines and benchmarks, we hope to accelerate the processing of challenging helical assemblies and unlock new structure-driven insights into amyloid disease pathology and therapeutic intervention strategies.

## 2. Approach

### 2.1. Processing guidelines for amyloids using *cryoSPARC*

**Figure 2:**
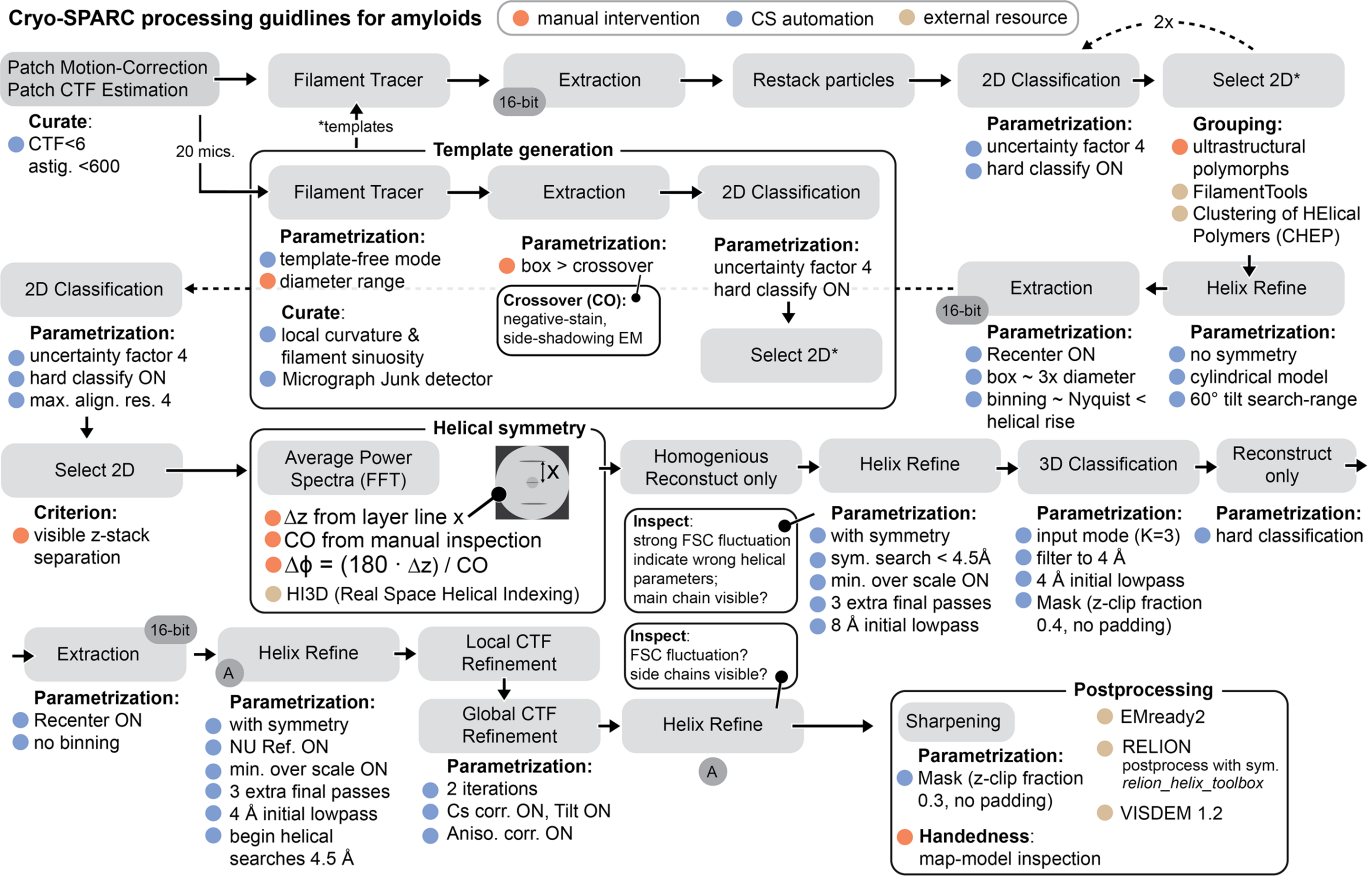
Processing guidelines for amyloids using *cryoSPARC*. Individual *cryoSPARC* job types are displayed in gray boxes, with recommended nondefault parameters marked by blue bullets. Steps requiring manual intervention or supplementary experiments are denoted by orange bullet points and external resources are denoted by tan bullet points.

The following approaches for reconstructing amyloids in *cryoSPARC* were identified through systematic parameter optimization and provide a foundation for target-specific adjustments, although universal applicability may vary. A comprehensive workflow is depicted in Fig. 2 ▸.

#### 2.1.1. Preprocessing and initial considerations

For image pre-processing, beam-induced motion was corrected using patch motion correction, followed by patch CTF estimation. Amyloids typically accumulate in thicker ice regions near grid hole edges, necessitating less stringent selection criteria than conventional single-particle analysis. We retained micrographs with CTF fits better than 6 Å and discarded those with astigmatism values exceeding 600 Å. In general, fewer high-quality micrographs generate better reconstructions than large datasets of non-ideal quality. Picking templates were generated using template-free filament tracing on 20 representative micrographs. The tracer identifies helical filaments as short, straight, partially overlapping segments using an approach adapted from *SPRING* (Huber *et al.*, 2018 ▸). These extracted segments serve as input for iterative helical real-space reconstruction (IHRSR; Egelman, 2007 ▸). Both *RELION* and *cryoSPARC* implement the IHRSR algorithm, segmenting filaments into overlapping short segments that can be averaged while preserving the ability to separate heterogeneous populations. This segmentation approach is feasible, but comes with limitations. Under ideal conditions (no Bessel overlap, well ordered straight filaments, adequate sampling), single projections of helical assemblies can contain sufficient information for reconstructing the asymmetric unit. However, in the presence of Bessel overlap or poorly sampled layer lines, this is not strictly true (Egelman, 2007 ▸).

#### 2.1.2. Filament tracing to 2D classification

Filament-tracing efficiency improved when diameters slightly below the actual filament dimensions were selected, combined with increased standard deviation values for Gaussian blur (0.4–0.5) to enhance filament contour prominence. To separate visually distinct polymorphs, the initial extraction box should exceed the approximate crossover distance, which can be estimated from high-defocus cryo-EM micrographs or negative-stain transmission electron microscopy pre-screening. For poorly performing picks, it is recommended to test a range of filament diameters on a small subset of micrographs, starting with diameters below the measured values.

Two rounds of 2D classification effectively generate well aligned class averages that enable the separation of different filament types. Optimal results were achieved using a large number of classes (*K* = 100) with an increased initial uncertainty factor (*e.g.* 4) and 40 expectation–maximization iterations, enabling hard classification during the final iteration. This approach effectively reduces per-class heterogeneity, as shown by high effective sample size (ESS) values approaching unity and Fourier ring correlation (FRC) values near the Nyquist frequency. If many classes show ESS values near 2, a larger number of classes (up to *K* = 200) may mitigate per-class heterogeneity, resulting in higher class quality. However, not all amyloids yield meaningful reconstructions due to harsh extraction protocols or compositional and conformational flexibility. In these cases, 2D classification results in low-contrast averages that lack protein-like features.

#### 2.1.3. Initial reconstruction generation and polymorphism management

Unlike *RELION*, *cryoSPARC* does not support initial model generation with helical priors using projection overlaps across entire helical crossovers (He & Scheres, 2017 ▸). Instead, *cryoSPARC* generates cylindrical starting models through its Helical Refinement job. Selecting homogeneous projections that span an entire crossover distance proves most efficient for initial model generation (Supplementary Fig. S1). Using smaller extraction boxes may produce mixed starting models from 2D classes of different polymorphs. Like *RELION*’s expectation–maximization optimizer, the initial volume must fall within the convergence search range to generate accurate final reconstructions (He & Scheres, 2017 ▸).

Separating inherent heterogeneity (polymorphism) requires manual grouping of matching 2D projections in *cryoSPARC*. Advanced tools such as *FilamentTools* for bi-hierarchical classification of filament 2D projections in *RELION*5 (Lövestam *et al.*, 2024 ▸) or *Clustering of Helical Polymers* (*CHEP*; Pothula *et al.*, 2021 ▸) are not yet available in *cryoSPARC*. Consequently, users must manually select class averages representing suspected polymorphs, with the inherent risk of false-positive selections.

The initial helical refinement should proceed without imposing axial or screw symmetry constraints, without limiting shifts along the helical axis and with maximum out-of-plane tilt searches set to 60°. It is recommended to inspect the reported final tilt distribution, expecting a centered tilt-range distribution within 0–20° bounds. Larger tilt ranges may indicate an incorrect reconstruction. Given the importance of the helical starting model, users are advised to generate several starting models with different sets of 2D class averages, followed by manual inspection. Large variations between reconstructions can indicate convergence to different local reconstruction minima without converging to the correct reconstruction solution. All of these reconstructions should be explored in downstream processing.

#### 2.1.4. Symmetry parameter determination

While large extraction boxes with high binning enable initial amyloid identification and rough separation of protomer-level polymorphs, helical segments should be re-extracted using smaller box sizes (approximately 300 Å or three times the amyloid diameter) with reduced binning to resolve β-stack separation. This approach typically matches the Nyquist frequency to values below the expected stack of many amyloids (approximately 4.8 Å). Of note, the stacking distance of adjacent cross-β-sheets is constrained by hydrogen-bond networks of the backbone amide and carbonyl, resulting in a tight range of 4.7–4.8 Å, visible in the average power spectrum of amyloid 2D class averages as strong layer lines (Almeida & Brito, 2020 ▸; Fig. 2 ▸). To select projections displaying visible stack separation, segments were re-extracted, recentered and subjected to 2D classification using maximum alignment resolution of 4 Å, an initial class uncertainty factor of 4 and hard classification enabled during the final iteration (Supplementary Fig. S1). In cases of a sharp FSC falloff near the resolution range of the rise (4–5 Å), the half-maps may be shifted along the *Z* axis. An established route to overcome *Z*-shifted half-maps is to manually align the half maps (for example in *ChimeraX*) and replace the shifted map in the *cryoSPARC* job directory prior to subsequent refinements (Lövestam & Scheres, 2022 ▸; Scheres, 2020 ▸).

The Average Power Spectra job type applied to well resolved projections enables determination of helical rise from prominent layer lines. By measuring the distance from the equator to the layer line of interest (*d* in pixels), the rise (Δ*z* in Å) can be calculated as Δ*z* = (box size)/(*d*) × 2 × (pixel size). Approximating helical twist (Δφ) requires the crossover distance, which must be estimated from low-pass-filtered micrographs or complementary methods such as negative-stain transmission electron microscopy or atomic force microscopy. The twist can then be calculated using Δφ = (Δ*z* × 180°)/(crossover distance).

While reciprocal-space measurements provide a robust approach for deriving screw parameters, real-space helical indexing tools such as *HI*3*D*, accessible through its web-based server (Sun *et al.*, 2022 ▸) offer alternatives by using projections of asymmetric helical reconstructions without requiring manual approximations. For improved *HI*3*D* accuracy, finer sampling parameters should be employed: angular steps of 0.5° and axial steps of 0.2 Å (Li, Muñoz Pérez *et al.*, 2025 ▸). Before proceeding to helical refinement, particle quality should be assessed using the ‘Homogeneous Reconstruction Only’ job with enforced helical rise applied to the selected particle subset. However, whenever using tools such as *HI*3*D* or *cryoSPARC*’s helical search utility, one must bear in mind that the reference volume from a helical refinement without symmetry may have itself already converged to an incorrect reconstruction. Subsequent refinements with helical parameters will often or always fail if the incorrect initial reconstruction is used as the starting point. In previous work, it was recommended that several initial reconstructions be generated and helical parameters identified using *HI*3*D*. Pursuing multiple parallel reconstructions with each starting model and the identified helical symmetry parameters can serve as a means of overcoming entrapment at local refinement minima (Egelman, 2024 ▸).

#### 2.1.5. Final amyloid reconstruction through refinements and classifications

To assess the accuracy of the approximated helical parameters, helical refinements should be performed with helical symmetry enabled, 8 Å initial low-pass filtering, per-particle scale minimization enabled and three additional final passes. The maximum out-of-plane tilt should be set to 60°. Symmetry searches should commence only at 4.5 Å resolution to maintain convergence toward separated cross-β-stacks. Users are advised to set helical symmetry searches for the rise very close to the expected value, using bounds of 4.6–4.9 Å, while helical twist values can be explored more broadly. Additionally, helical parameter searches can be inspected by the *cryo­SPARC* job log output of the Helical Symmetry Error Surface. A discrete high-confidence signal indicates convergence to the correct helical parameters, while streaks across the length of the entire axis indicate unresolved parameters.

Strong fluctuations in FSC curves with high discrepancies between tight and corrected FSC curves for auto-masking may indicate incorrect helical symmetry for the tau reconstructions (Supplementary Figs. S1*e* and S1*f*). In contrast, no such fluctuations are present for the ALys reconstruction (Supplementary Fig. S1*b*). Additionally, a strong cross-correlation falloff around 4.8 Å indicates axial misalignments between the half-maps, which are often visible upon manual inspection of the half-maps. This register shift can be corrected by uploading a shift-corrected combined map using the volume maximum command from *ChimeraX* to *cryo­SPARC*.

The user should also pay special attention to the selection of suitable masks. Fibrils extend beyond the extraction box and require different approaches for masking. The recent *CubeNTube* extension for *ChimeraX* (https://doi.org/10.5281/zenodo.18754999) assists users in generating wide cylindrical masks during initial reconstructions. Dynamic masking should be disabled at this stage. After obtaining a reasonable reconstruction, users should apply a low-pass filter to their reconstruction using Gaussian filters (volume Gaussian in *ChimeraX*) and upload this filtered volume to *cryoSPARC* to generate a soft mask using default padding parameters. To reduce misalignment from increased fibril curvature near the box edges, the *z*-clip fraction can be reduced to 0.4 times the total box size, but other values for more rigid or curved fibrils may be tested when setting auto-masking parameters. Strict sinuosity and curvature cutoffs during inspection of traced fibrils may help to reduce fibril misalignment.

For samples where there is substantial compositional or conformational heterogeneity, 3D classification without alignment may facilitate particle separation into distinct structural classes, enabling symmetry search convergence on subsets to achieve meaningful reconstructions. Reported resolution estimates at this processing stage are unlikely to reflect the actual resolution of the structures, and reconstructions should be critically examined qualitatively for main-chain visibility and stack separation. Classification using input mode with three classes (*K* = 3), filtered to 4 Å with a low-pass filter at 4 Å and focused masking (*z*-clip fraction of 0.4, no extra padding) may successfully separate low-quality particles or distinct polymorphs. A ‘Reconstruction Only’ job with hard classification enabled and enforced rise values should be performed to inspect reconstructions and select consensus structures for further processing.

The selected particle stack should then be re-extracted and recentered without binning, followed by another round of helical refinement with symmetry searches enabled (starting at 4.5 Å), 4 Å low-pass filtering, non-uniform refinement enabled for three additional final passes and per-particle scale minimization enabled. Local and global CTF refinement should be attempted, as these jobs may further enhance reconstruction quality. However, after each CTF refinement step, subsequent helical refinements should be performed to evaluate improvements. The resulting reconstructions require careful manual inspection, comparing observed density quality against prior reconstructions, rather than relying on the reported FSC-based resolution estimates, to ensure meaningful structural interpretation.

Global CTF refinement with three iterations should include both tilt and spherical aberration correction. In our testing, reference-based motion correction was not performed since not all selected datasets included movie data, although it may provide further resolution improvements where movie frames are available. Finally, reconstructions can be post-processed using *B*-factor sharpening within *cryoSPARC*, employing masks with *z*-clip fractions of 0.3 and no additional padding. Alternatively, *EMready*2 (Cao *et al.*, 2025 ▸) or *VISDEM*1.2 (https://github.com/gschroe/visdem) can be used to improve reconstruction interpretability and facilitate model building. While *EMready*2 performs well as a universal non-*B*-factor sharpening tool, *VISDEM*1.2 supports the application of helical symmetry, further aiding model building for amyloids.

Most amyloids are deposited in structural databases as left-handed assemblies (exhibiting negative twist values), but absolute handedness must be experimentally verified rather than inferred. This is critical because cryo-EM projection images are inherently ambiguous with respect to handedness: a right-handed helix viewed from one direction produces the same 2D projection as a left-handed helix viewed from the opposite direction. This fundamental ambiguity means that helical reconstructions can converge to either the correct or incorrect handedness with equally good agreement with the experimental data. At sufficiently high resolution (typically better than 3 Å), backbone traces can directly inform handedness determination through visualization of peptide backbone chirality. However, for reconstructions with lower interpretability, techniques such as platinum side-shadowing transmission electron microscopy or atomic force microscopy could be used to discern the handedness of the fibril (Kollmer *et al.*, 2019 ▸). Even by integrating these approaches, there will remain a degree of ambiguity regarding handedness, and this caveat should be explicitly stated with the description of the structure. Comparison of helical parameters and structural features with database entries, such as those in the Amyloid Atlas (Sawaya *et al.*, 2021 ▸), can also facilitate both model building and handedness verification. However, even deposited reconstructions may have an incorrect handedness, and thus researchers should remain critical about when and how to assign absolute handedness.

## 3. Results

### 3.1. Reconstructing amyloids to high resolution using *cryoSPARC*

To establish robust processing guidelines for reconstructing amyloids in *cryoSPARC*, six EMPIAR datasets were selected for systematic evaluation. These include five *RELION*-processed depositions and one recent *cryoSPARC*-processed deposition: *ex vivo* paired-helical filament (PHF-type) tau, recombinant intermediate-amyloid tau (MIA-type), cardiac transthyretin amyloids (ATTR), recombinant β2-microglobulin variant V27M, *ex vivo* lysozyme D87G and *ex vivo* light-chain Lambda6 amyloids (Table 1 ▸). The datasets were selected to represent the diversity of amyloid diseases, origins (*in vitro* versus *in vivo*), helical symmetries, data quality, availability and dataset sizes.

**Figure 3:**
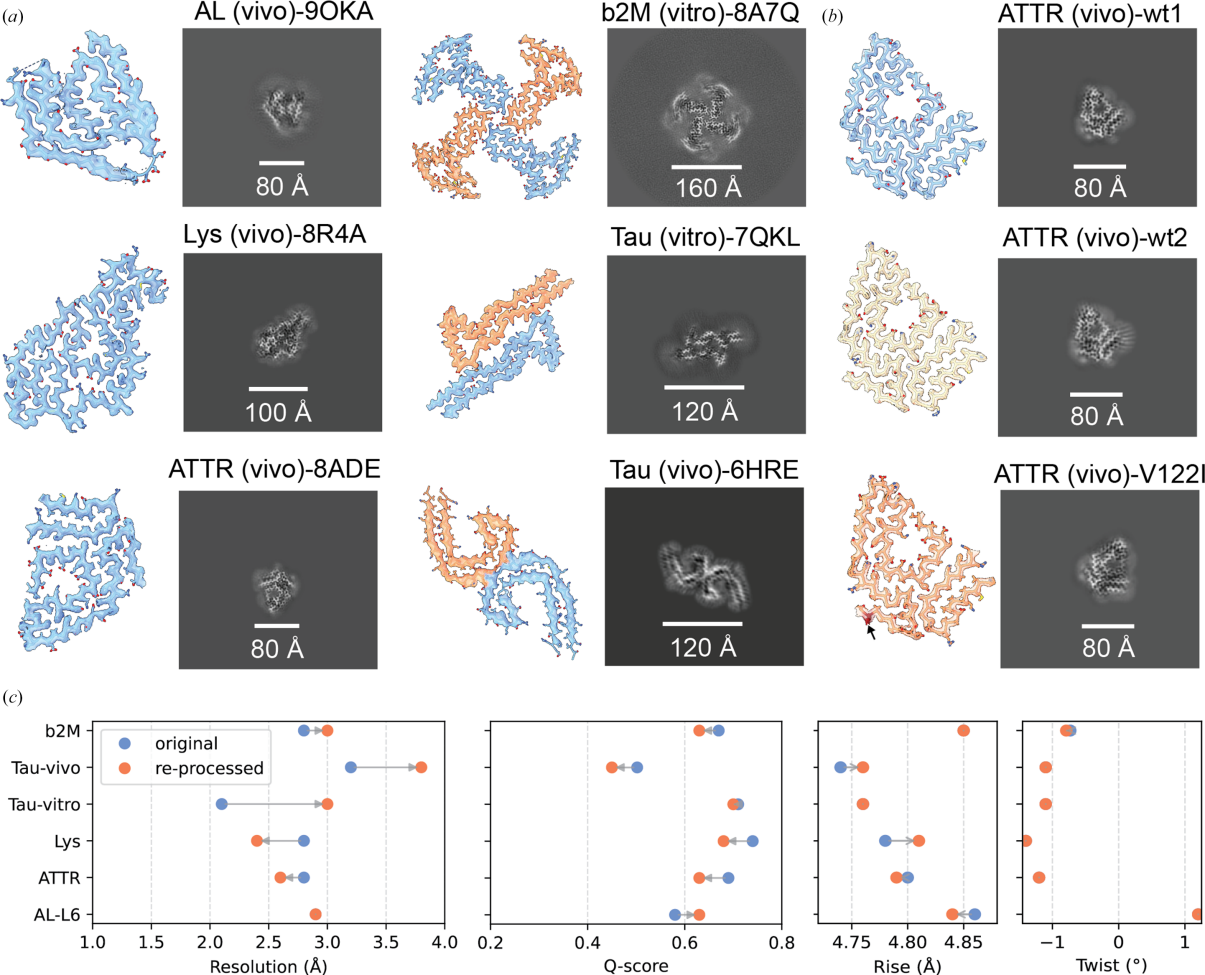
Performance of amyloid processing using *cryoSPARC*. (*a*) Map–model fits and *xy* slices with scale bars for refined reconstructions of amyloid datasets from EMPIAR. Light-chain amyloids (AL; PDB entry 9oka), β_2_-microglobulin variant V27M (b2m; PDB entry 8a7q), lysozyme variant D87G (Lys; PDB entry 8r4a), *ex vivo* PHF-type tau (PDB entry 6hre), transthyretin amyloids (ATTR; PDB entry 8ade) and recombinant tau MIA-type amyloids (PDB entry 7qkl). Single-protofilament polymorphs are shown in blue and multi-protofilament polymorphs in blue and orange. (*b*) Cardiac ATTR reconstructions from our previous work showing two wild-type transthyretin samples and familial variant V122I (indicated by an arrow). (*c*) Validation of re-processed EMPIAR datasets (orange) compared with the original depositions (blue, from Table 1 ▸) with resolutions estimated according to an FSC 0.143 cutoff, *Q*-scores were calculated with *ChimeraX* and helical symmetry parameters.

Following the processing guidelines described above, light-chain, transthyretin, lysozyme, *in vitro* tau and β_2_-microglobulin amyloids were successfully reconstructed with high confidence, showing well resolved side chains (Fig. 3 ▸*a*, Supplementary Fig. S1*d*). However, the *ex vivo* tau dataset failed to yield a correct reconstruction (Fig. 3 ▸*a*), highlighting the common problem of convergence to incorrect local refinement minima (He & Scheres, 2017 ▸). While β-stack separation for *ex vivo* PHF tau was clearly observable in 2D class averages (Supplementary Fig. S1*f*), refinement did not converge on correct helical parameters, resulting in an incorrect reconstruction exhibiting signs of overfitting (Supplementary Fig. S1*f*). Despite extensive iterative refinement attempts with various starting models and symmetry parameters, we were unable to obtain a reliable reconstruction of the PHF tau structure using *cryoSPARC*. This limitation likely reflects the challenges inherent to complex polymorphic structures rather than fundamental inadequacies of the platform, suggesting that further methodological development could enable successful reconstruction of such challenging targets.

In our recent work, we characterized cardiac ATTR filaments from three different patients (wild-type and familial V122I variant transthyretin), linking amyloid deposits to microangiopathy and capillary occlusion (Donnelly *et al.*, 2026 ▸). These amyloids share the common spearhead fold characteristic of transthyretin amyloids, which we reconstructed to high resolution using the presented workflow (Fig. 3 ▸*b*, Table 1 ▸). Thus, our processing guidelines enabled the meaningful reconstruction of eight out of nine selected datasets in *cryoSPARC*.

Notably, many amyloids exhibit structural polymorphism (Annamalai *et al.*, 2016 ▸). Based on visible protofilaments in 2D class averages, no oligomer-level polymorphism was observed for β2-microglobulin, lysozyme, ATTR or tau (Supplementary Fig. S1). Consistent with the original work, the processed light-chain amyloids assemble into single- and double-protomeric arrangements (Hicks *et al.*, 2026 ▸), but only single-chain amyloids yielded successful reconstructions. Reprocessing identified a double-filament polymorph arrangement in light-chain amyloids (2D class averages in Supplementary Fig. S1*a*), which did not produce a convincing reconstruction, potentially because we failed to identify the correct helical symmetry. Of note, a double-filament polymorph arrangement was previously reported for a different Lambda6 germline light chain (IGLV6-57 AL), for which the authors suggested a unique rotational symmetry inherent to amyloids, with protomers being related by a 180° rotation perpendicular to the fibril axis (Bassett *et al.*, 2024 ▸).

### 3.2. Validating helical reconstructions using *cryoSPARC*

Helical reconstructions are inherently susceptible to artifacts and subsequent misinterpretation due to the imposition of high-order helical and axial symmetry constraints that inflate the reported resolutions. A recent preprint reported errors in approximately 14% of helical reconstructions deposited in the EMDB, identified through real-space helical indexing and manual data curation (Li, Muñoz Pérez *et al.*, 2025 ▸). Given the prevalence of such biases, FSC-based resolution estimation alone cannot reliably assess helical reconstruction quality (Fig. 3 ▸*c*, Supplementary Fig. S1) and should not serve as the sole validation criterion. Therefore, helical symmetry correctness must be supported by comprehensive map–model assessment, including clear cross-β-stack separation, continuous backbone traces, side-chain resolvability and proper stereochemistry (He & Scheres, 2017 ▸).

To quantify the interpretability of our amyloid reconstructions, *Q*-scores (Pintilie *et al.*, 2020 ▸) were calculated using *ChimeraX* and compared with original deposited data using corresponding identifiers from Table 1 ▸. *Q*-scores were calculated for single cross-β-stacks and plotted in Fig. 3 ▸(*c*) (detailed values are given in Supplementary Table S1). While most reconstructed amyloids support the fitted atomic models with *Q*-scores above 0.6, the original *RELION* reconstructions yielded higher interpretability values. The reprocessed light-chain AL-L6 from *cryoSPARC* showed modest improvement compared with the original deposition. Comparison of applied helical symmetry parameters reveals minor discrepancies in helical rise values, potentially contributing to reduced map interpretability. Other established signs of convergence of the refinement to an inaccurate local minimum (Lövestam & Scheres, 2022 ▸; Scheres, 2020 ▸) are discontinuous density of the backbone trace, negative density between cross-β-strands and nonprotein fluctuations in local density intensity, which we observed for the PHF tau polymorph (Fig. 3 ▸*a* and Supplementary Fig. S1).

## 4. Discussion

In our previous work on *ex vivo* ATTR amyloids, we demonstrated the capability of *cryoSPARC* to reconstruct amyloids from complex tissue extracts to high resolution (Fig. 3 ▸*b*). This success motivated the development of systematic processing guidelines for amyloid reconstruction using *cryoSPARC* as a viable alternative to *RELION*. Following our ATTR work, we reprocessed deposited amyloid datasets using our established workflow, successfully reconstructing five of six selected amyloid datasets to high resolution; only one dataset failed to achieve suitable resolution for model building, since it converged onto a local but incorrect minimum.

Notably, our new processing workflow, available as import-ready .json files (https://github.com/schaefer-jh/CS-amyloids/), achieved 2.4 Å global resolution for ALys amyloids (Fig. 3 ▸*a*, Supplementary Fig. S1) in approximately 12 h using three GPUs for multi-GPU processing of ∼2000 movies. By integrating our recently published deep neural network *CryoSift* (Schäfer *et al.*, 2025 ▸), which automatically selects suitable 2D class averages through its integrated mode with *cryoSPARC* tools, unsupervised high-throughput processing of amyloids with known helical symmetry becomes feasible in *cryo­SPARC*, potentially accelerating future drug-development efforts. While many of the presented nondefault processing parameters facilitate reconstruction of homogeneous amyloids and protofilament-level polymorphs in *cryoSPARC*, reliable classification of polymorphs at the peptide level remains challenging.

We also revisited previous use cases of *cryoSPARC* for amyloid reconstructions, identifying several commonalities that are of interest and might be worth exploring. All four available processing routines (PSMα1, EMD-43835; AL, EMD-70557; FKP, EMD-53305; FapC, EMD-49649) used *cryoSPARC*’s filament tracer over the *TOPAZ* picker and combined binned 2D classification to generate optimized templates for the filament tracer. If polymorphs were present, the authors used large box sizes to manually classify segments into distinct fibril types (PSMα1, AL and FapC). The use of *ab initio* model generation and iterative 3D classification using the input mode was also successfully utilized, but this route has been less reproducible and was not selected for the presented workflow.

To enhance *cryoSPARC*’s capabilities as a complementary platform for amyloid reconstruction, several targeted improvements would expand its utility alongside existing tools such as *RELION*. Key improvements include implementing noncylindrical starting-model generation using projection-level information from overlapping 2D class averages, such as *relion_helix_inimodel*2*d* in *RELION* (He & Scheres, 2017 ▸) or *denovo*3*d* in *Helicon* (Li, Zhang *et al.*, 2025 ▸). Coupled with automatic clustering of 2D class averages, analogous to *RELION*’s *FilamentTools* (Lövestam *et al.*, 2024 ▸), this model-generation approach would yield more homogeneous particle subsets, which would help mitigate the common problem of convergence to local refinement minima by narrowing the search range for initial model generation while reducing user-dependent bias/error in class selection (He & Scheres, 2017 ▸; Lövestam & Scheres, 2022 ▸). Another approach would be to generate multiple initial helical reconstructions without imposing symmetry, then use existing tools such as *HI*3*D* or *cryoSPARC*’s symmetry search-utility tool to identify helical parameters for a helical refinement (Egelman, 2024 ▸). A streamlined adaptation of this workflow, supporting multi-class helical reconstructions analogous to its *ab initio* reconstruction for globular proteins, would be an impactful addition to a future version of *cryoSPARC*.

While large-scale polymorphs such as single and double protofilaments of light-chain amyloids (Supplementary Fig. S1) were readily classified at the projection level, previous studies on light-chain Lambda6 (Bassett *et al.*, 2024 ▸) demonstrated that double protofilaments contain additional peptide-level morphologies that require further 3D classification. These polymorphs and structural intermediates are likely to play crucial roles in amyloid disease progression (Tycko, 2015 ▸) and have been successfully dissected using time-course cryo-EM experiments (Lövestam *et al.*, 2024 ▸). Separation and high-resolution reconstruction of *ex vivo* amyloid mixtures is essential for therapeutic development; however, *cryoSPARC* currently lacks 3D classification with helical rise and twist optimization, making separation of unique but similar filament types difficult or impossible. Implementing classification routines with helical symmetry optimization, both with alignment in Heterogeneous Refinement and without alignment in 3D Classification jobs, would enable broader access to amyloid structure determination using* cryoSPARC*.

Our study presents the current capabilities and limitations of *cryoSPARC* for amyloid reconstruction using optimized non­default parameters. The established guidelines should enable the reconstruction of diverse amyloids, spanning functional and pathogenic assemblies; however, further optimization of amyloid-specific software is needed to advance *cryoSPARC*’s potential as a universal platform for helical reconstructions. These community-driven guidelines aim to improve the accessibility and reproducibility of amyloid structure determination, enabling broader participation in amyloidosis research and accelerating the development of therapeutic interventions.

## 5. Methods

### 5.1. Data selection and curation

For database searches, the EMDB REST API (https://www.ebi.ac.uk/emdb/api/) was utilized to automatically extract the *Q*-score and resolution values for matching EMDB entries of amyloids reported by the Amyloid Atlas (Sawaya *et al.*, 2021 ▸). For testing and validating our processing guidelines in *cryoSPARC*, we reprocessed the amyloid reconstructions from six EMPIAR datasets, listed in Table 1 ▸.

### 5.2. Amyloid processing

All datasets were processed in *cryoSPARC* v.4.6-7 on NVIDIA Tesla L4 GPUs and Intel Xeon CPU E5-2698 v4 with up to four GPUs for multi-GPU CS job types. Processing of EMPIAR datasets was performed following the established guidelines shown in Fig. 2 ▸ and described in Section 2. A comprehensive validation of the individual datasets is shown in Supplementary Fig. S1 and Supplementary Table S1. Results were visualized using *ChimeraX* (Pettersen *et al.*, 2021 ▸) and *ggplot* in Python. Pseudo-screw symmetry in tau amyloids (*P*2_1_) was converted to a *C*2 point group for ease of visualization by multiplying the rise value by two and subtracting 180 from the twist value.

## Supplementary Material

Supplementary Figure and Table. DOI: 10.1107/S2053230X26003675/ih5010sup1.pdf

## Figures and Tables

**Table 1:** EMPIAR datasets for processing in cryoSPARC * denotes our own work, processed in *cryoSPARC* (v.4.6-7). Right-handedness is denoted with positive twist values and left-handedness with negative twist values.

EMPIAR	EMDB code	*d*† (Å)	PDB code	Protein (source)	No. of micrographs	Å per pixel	Dose (e Å^−2^)	Cs (mm) (keV)	Δ*z* (Å)	Δφ (°)	Symmetry	CO‡ (Å)
10940	EMD-14046	2.1	7qkl	Tau (*ex vitro*)	331	0.824	40	2.7 (300)	2.38	179.4	*C*1	778
10230	EMD-0259	3.2	6hre	Tau (*ex vivo*)	507	1.15	60	2.7 (300)	2.37	179.5	*C*1	776
11785	EMD-18883	2.8	8r4a	Lysozyme (*ex vivo*)	2013	1.04	49	2.7 (300)	4.78	−1.4	*C*1	614
11383	EMD-15224	2.8	8a7q	β2m-V27M	611	0.94	43	2.7 (300)	4.85	−0.73	*C*2	1196
12815	EMD-70557	2.9	9oka	AL (*ex vivo*)	7076	0.90	40	2.7 (200)	4.86	1.2	*C*1	729
12217	EMD-15361	2.8	8ade	ATT (*ex vivo*)	1943	1.04	45	2.7 (300)	4.80	−1.2	*C*1	720
12909*	EMD-7195	3.2	9px6	ATTR (*ex vivo*) wt1	1910	0.94	50	2.7 (200)	4.85	−1.26	*C*1	693
12911*	EMD-71960	3.4	9px7	ATTR (*ex vivo*) wt2	2426	0.94	50	2.7 (200)	4.85	−1.28	*C*1	682
12912*	EMD-71962	3.0	9px9	ATTR V122I (*ex vivo*)	2326	0.94	50	2.7 (200)	4.85	−1.24	*C*1	704

† Resolution.

‡ Helical crossover. CO = [180° × Δ*z* (Å)]/|Δφ (°)|.

## Data Availability

All reprocessed datasets from the Electron Microscopy Public Image Archive (EMPIAR) were deposited in the Electron Microscopy Data Bank (EMDB). The datasets from Supplementary Table S1 are available using the following EMDB accession numbers with their respective EMPIAR IDs: ATTR wt1 (EMD-71953, EMPIAR-12909), ATTR wt2 (EMD-71960, EMPIAR-12911), ATTR V122I (EMD-71962, EMPIAR-12912), AL-L6 (EMD-72157, EMPIAR-12815), ATTR (EMD-72158, EMPIAR-12217), b2M V27M (EMD-72159, EMPIAR-11383), Lys D87G (EMD-72161, EMPIAR-11785), tau MIA (EMD-72164, EMPIAR-10940) and tau PHF (EMD-72174, EMPIAR-10230). An import-ready .json workflow for *cryoSPARC* (generated in v.4.7.0) is available at https://github.com/schaefer-jh/CS-amyloids/ and can be used per the developers’ instructions (https://guide.cryosparc.com/application-guide-v4.0+/workflows).
