# Supplementary material for: Helical reconstruction of amyloids in cryoSPARC
Source: Acta Crystallogr F Struct Biol Commun. 2026 Jun 9;82(Pt 7):252–61. doi: 10.1107/S2053230X26003675 (PMC13335050; doi:10.1107/S2053230X26003675)
Supplement: Supplementary file 1 [file f-82-00252-sup1.pdf]

STRUCTURAL BIOLOGY COMMUNICATIONS

Volume 82 (2026)

Supporting information for article:

Helical reconstruction of amyloids in *cryoSPARC*

Jan-Hannes Schaefer, Robert T. O'Neill, Joseph P. Donnelly, Evan T. Powers, Jeffery W. Kelly and Gabriel C. Lander

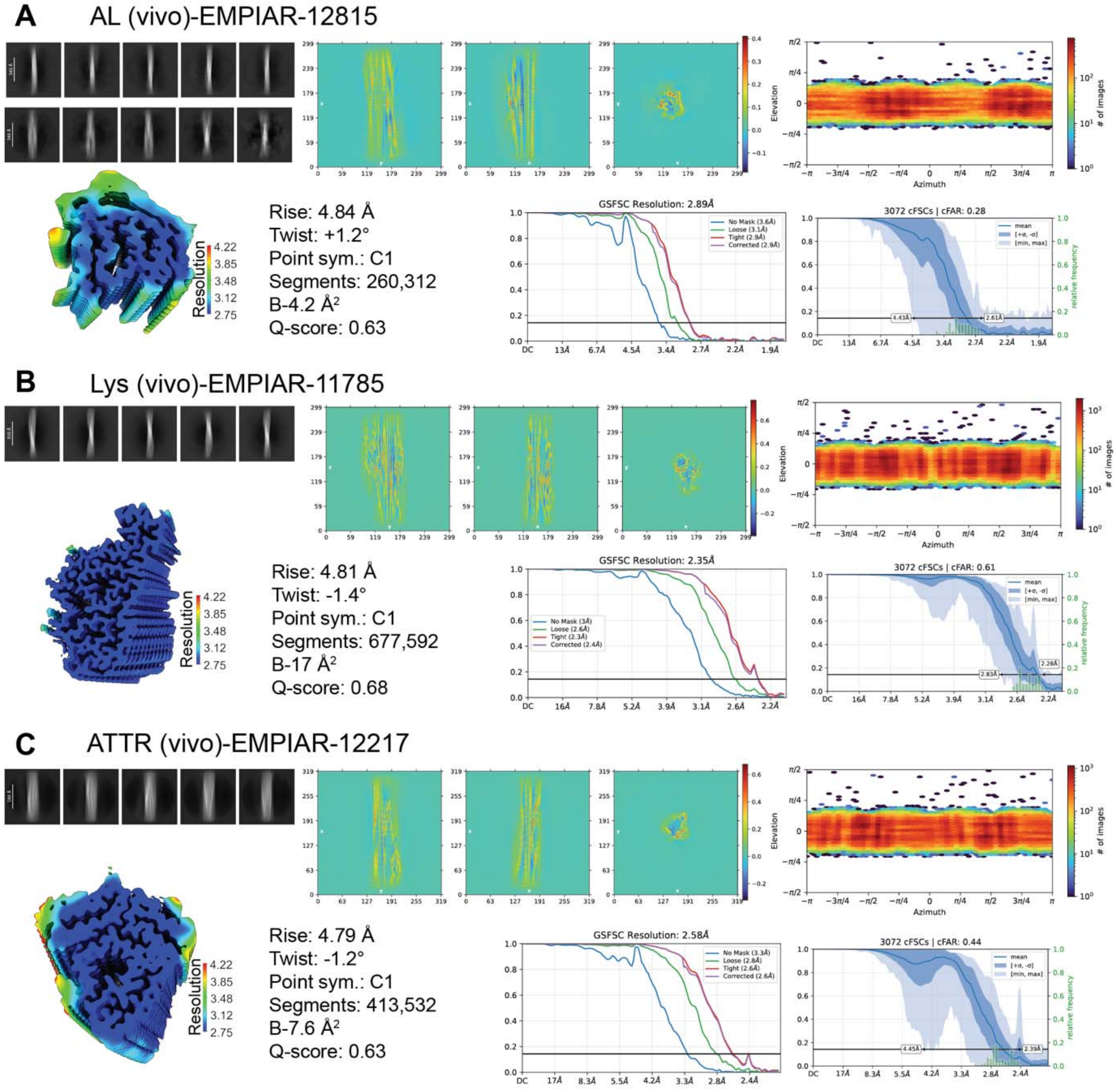

Figure S1 Validation of amyloid reconstructions using cryoSPARC. Comprehensive validation for each EMPIAR dataset includes 2D class averages, real-space slices along major orthogonal planes (zy, zx, xy), viewing directional distribution, local resolution maps (color-coded consistently across all datasets), resolution estimated using gold-standard FSC (cutoff at 0.143), and cFAR reports. (**A**) Light-Chain amyloids, (**B**) Lysozyme amyloids and (**C**) Transthyretin amyloids.

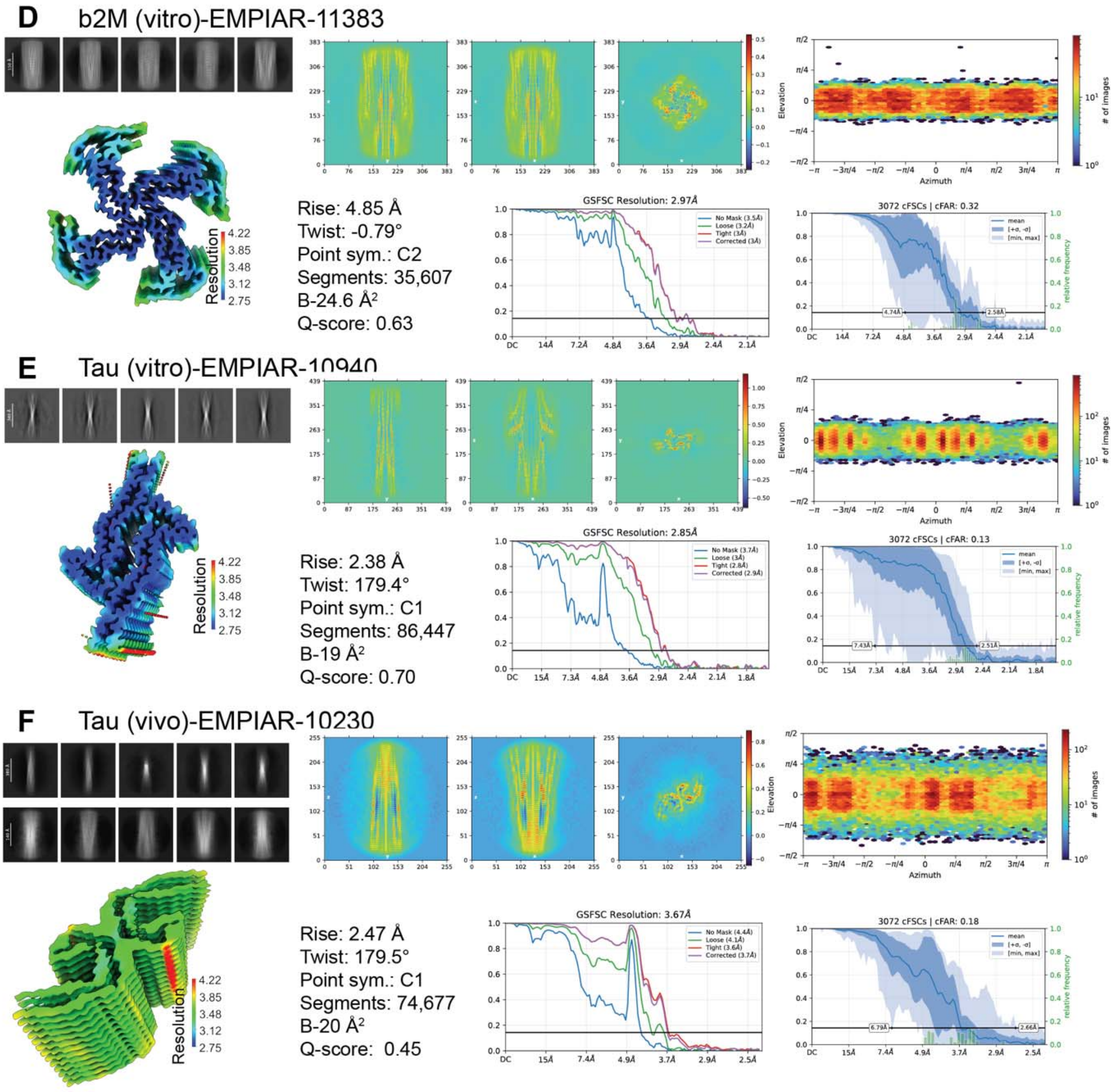

Figure S1 (continued). (**D**) Beta-2-macroglobulin amyloids (**E**) in vitro Tau amyloids and (**F**) in vivo Tau amyloids.

Table S1 Cryo-EM data collection and image processing of amyloids. (NA, not applicable. -, data not available).

	**ATTR wt1**	**ATTR wt2**	**ATTR V122I**	**AL-L6**	**ATTR**	**b2M V27M**	**Lys D87G**	**Tau MIA**	**Tau PHF**
EMDB ID	71953	71960	71962	72157	72158	72159	72161	72164	72174
EMPIAR ID	12909	12911	12912	12815	12217	11383	11785	10940	10230
**Data Collection**									
Microscope	Arctica	Arctica	Arctica	Arctica	Krios	Krios	Krios	Krios	Krios
Camera	Falcon 4i	Falcon 4i	Falcon 4i	Falcon 4i	K2 Quantum	Falcon 4i	K2 Quantum	Falcon 4i	K2 Summit
Magnification (nominal)	150,000	190,000	190,000	130,000	130,000	130,000	130,000	96,000	105,000
Voltage (keV)	200	200	200	200	300	300	300	300	300
Total dose ($e^-/Å^2$)	50	50	50	40	45	43	49	40	60
Exposure rate (e^-/px/s)	11.07	11.07	10.81	10.82	3.1	7.6	5.9	-	13.8
Frames per movie	1,395	1,395	1,386	-	40	1,442	40	-	50
EER Fractions	40	40	40	40	NA	40	NA	40	NA
Pixel size (Å/px)	0.94	0.94	0.94	0.9	1.04	0.94	1.04	0.824	1.15
Defocus range (µm)	-2.0 to -0.8	-2.0 to -0.8	-2.0 to -0.8	-0.8 to -2.5	-1.2 to -2.5	-1.3 to -2.5	-1.0 to -2.0	-1.0 to -3.5	-1.7 to -2.8
Recorded movies	3,289	6,232	6,063	7,076	1,943	611	2,013	331	507
**Image Processing**									
Processing software	CS (v.4.6)	CS(v.4.6)	CS(v.4.6)	CS(v.4.7)	CS(v.4.7)	CS(v.4.7)	CS(v.4.7)	CS(v.4.7)	CS(v.4.7)
Final particle images	171,067	231,893	335,693	260,312	413,532	35,607	677,592	86,447	74,677
Symmetry imposed	C1	C1	C1	C1	C1	C2	C1	C1	C1
Helical twist (°)	-1.26	-1.28	-1.24	1.2	-1.2	-0.79	-1.4	179.4	179.4
Helical rise (Å)	4.85	4.85	4.85	4.84	4.79	4.85	4.81	2.38	2.39
Res. (FSC 0.143) (Å)	3.2	3.4	3.0	2.9	2.6	3.0	2.4	2.9	3.7
Local res. range (Å)	2.7-4.9	3.0-5.2	2.6-4.8	2.6-4.4	2.4-4.5	2.6-4.7	2.3-2.8	2.5-7.4	2.7-6.8
cFAR	0.31	0.27	0.28	0.28	0.44	0.32	0.61	0.70	0.45
Sharpening B-factor ($Å^2$)	-46	-68	-36	-4	-8	-25	-17	-18	-20
